# Comparison of the immune response to vaccination with pigeon circovirus recombinant capsid protein (PiCV rCP) in pigeons uninfected and subclinically infected with PiCV

**DOI:** 10.1371/journal.pone.0219175

**Published:** 2019-06-28

**Authors:** Tomasz Stenzel, Daria Dziewulska, Marcin Śmiałek, Bartłomiej Tykałowski, Joanna Kowalczyk, Andrzej Koncicki

**Affiliations:** Department of Poultry Diseases, Faculty of Veterinary Medicine, University of Warmia and Mazury in Olsztyn, Olsztyn, Poland; Sun Yat-Sen University, CHINA

## Abstract

Infections with immunosuppressive pigeon circovirus (PiCV) pose the most severe health problem to the global pigeon breeding. The vaccination with immunogenic PiCV recombinant capsid protein (PiCV rCP) is a potential tool for disease control. Because of the high prevalence of PiCV asymptomatic infections, the subclinically infected pigeons will be vaccinated in practice. The aim of this study was to answer a question if vaccination of asymptomatic, infected with PiCV pigeons induces a similar immune response to PiCV rCP as in uninfected birds. One hundred and twenty 6-week-old carrier pigeons were divided into 4 groups (2 groups of naturally infected and uninfected with PiCV individuals). Birds from groups V and V1 were vaccinated twice with PiCV rCP mixed with an adjuvant, whereas pigeons from groups C and C1 were immunized with an adjuvant only. The expression of genes encoding IFN-γ, CD4, and CD8 T lymphocyte receptors; the number of anti-PiCV rCP IgY-secreting B cells (SBC) and anti-PiCV rCP IgY were evaluated 2, 21, 39 and 46 days post vaccination (dpv). Study results showed that the expression of CD8 and IFN-γ genes was higher in both groups of infected pigeons than in the uninfected birds, irrespective of vaccination. In the uninfected birds, the expression of these genes was insignificantly higher in the vaccinated pigeons. The anti-PiCV rCP IgY-SBC were detected on 2 and 23 dpv and seroconversion was noted on 23 and 39 dpv in V and V1 groups, respectively. In the light of the results obtained, it could be concluded that pigeon circovirus recombinant capsid protein elicits the immune response in both naturally infected and uninfected pigeons, but its rate varies depending on PiCV infectious status. The infection with PiCV masks the potential cellular immune response to the vaccination with PiCV rCP and leads to the suppression of humoral immunity.

## Introduction

Pigeon circovirus (PiCV) is classified in the genus *Circovirus* of the *Circoviridae* family [1]. It is a small non-enveloped single-stranded circular DNA virus with approximately 2030 base pairs (bp) ambisense genome [2]. The genome of PiCV contains at least two major open reading frames (ORFs). Located on the virion sense strand ORF, V1, encodes the replicase protein (Rep protein) which is involved in rolling circle PiCV DNA replication. Located on the complementary sense strand ORF, C1, encodes the viral capsid protein (Cap protein, CP) [1, 2]. The CP of circoviruses has been documented to exhibit antigenic properties, as confirmed in the case of porcine circovirus genotype 2 (PCV2), psittacine circovirus, and pigeon circovirus [3–5].

Like other circoviruses, PiCV is an immunosuppressive factor. Infection with this virus leads to the atrophy of the immune system organs and to lymphocyte apoptosis [6–8]. Pigeons immunosuppressed by PiCV infection are predisposed to concomitant infections with other viruses (pigeon herpesvirus) or bacteria, like *Escherichia (E*.*) coli* and *Chlamydia (C*.*) psittaci* [9–11]. The combination of various immunosuppressive factors (PiCV infection and stress associated with trainings and racing of young pigeons) with accompanying infections leads to a clinical complex disease called young pigeon disease syndrome (YPDS) [9, 12, 13]. The type and intensity of clinical symptoms of YPDS are correlated with the type of the confounding factor. Initial symptoms are relatively non-specific, but after 2–3 days increased thirst and regurgitation from the crop is usually noticed. Crops are often filled with large volumes of water combined with mucus and refluxed duodenal contents. Other symptoms include diarrhea, apathy, feather ruffling or reluctance to training. Those birds are disqualified from racing [12]. For the reason of a very high global prevalence of PiCV infections approximating 70%, the YPDS is currently the biggest health issue in pigeon breeding [10, 12, 14, 15]. The global spreading of PiCV infections in pigeon population is probably due to pigeon racing and intercontinental trade [13].

The asymptomatic infections with this virus are quite common and approximate 40% [10, 16–19]. Their high prevalence in reproductive pigeons poses problems with disease control. The laboratory diagnosis is limited only to screening for PiCV genetic material with molecular methods [16]. The possibility of detecting anti-PiCV antibodies in subclinically infected pigeons has been described as well [19]. Because the laboratory culture of PiCV has so far proved unsuccessful, a scientific project was designed to develop an alternative method for obtaining an antigen which is recombinant capsid protein of pigeon circovirus (PiCV rCP). This protein could be used as an antigen in a sub-unit vaccine against this virus. A previous study has revealed PiCV rCP to be immunogenic to pigeons and to stimulate both cell-mediated and humoral immunity [5]. Due to high prevalence of PiCV asymptomatic infections [18, 19], it is very likely that the subclinically infected pigeons will be vaccinated in practice. Bearing in mind the potential immunosuppressive effect of PiCV, it is important to compare the immune response to PiCV rCP in pigeons asymptomatically infected with PiCV (natural infection) to that in the uninfected pigeons, before the protectivity of the vaccine is tested. The aim of this study was, therefore, to answer a question if subclinically infected with PiCV pigeons would develop a similar immune response to PiCV rCP to that developed by the uninfected birds.

## Materials and methods

### Ethical statement

This study was carried out in strict accordance with the Act of 21 January 2005 on animal experimentation and the Regulation of the Minister of Science and Information Technology of 29 July 2005 on the National Committee for Animal Experimentation. The research protocol was approved by the Local Ethics Committee on Animal Experimentation at the University of Warmia and Mazury in Olsztyn (Authorization No. 64/2014). The birds were euthanized by intravenous administration of pentobarbital sodium at 70 mg/1 kg BW (Morbital, Biowet Puławy, Poland) after premedication by intramuscular injection of butorphanol tartrate at 4 mg/1 kg BW (Torbugesic, Zoetis, USA). The researchers made every effort to minimize the suffering of birds.

### PiCV recombinant capsid protein (PiCV rCP)

The *cap* sequence of the pigeon circovirus representing the most common in the world genotype A was selected for the production of the PiCV recombinant capsid protein. Simultaneously, the selected isolate (PiCV PL_53, GenBank Acc. No.: KF738860.1) is the most divergent representative of its clade [18]. The recombinant capsid protein was expressed in *Escherichia (E*.*) coli* strain BL21 Star (DE3) chemically competent cells (Thermo Scientific, USA) according to the method described in our previous study [19].

### Vaccine preparation

Each vaccination dose (20μg of PiCV rCP/ pigeon) was prepared individually by mixing a volume of protein equivalent for an immunization dose with PBS (phosphate-buffered saline) (Sigma Aldrich, Germany) up to the volume of 200 μL. Next, it was mixed with 200 μL of an oil-based adjuvant (Montanide ISA71 R VG) provided by Seppic (France). The mixed equal volumes of the adjuvant and PBS (400 μL in total) were used as a vaccination control.

### Pigeons

Prior to the experiment, three naturally infected with PiCV flocks of pigeons were tested in order to select birds infected with a separate genotype of pigeon circovirus than the isolate used to produce PiCV rCP. To this end, the CP encoding gene was amplified with the method described previously [15]. All of the obtained isolates belonged to genotype A, additionally two of the three investigated PiCV isolates showed strong homology with the PiCV PL_53 isolate used for the production of the vaccine. Therefore the birds were selected for experiment from the flock infected with the most genetically different from vaccine strain PiCV_2T isolate (88% and 92% nucleotide and amino acid homology, respectively) (Acc. No.: MK994767) (S1 Fig). Next, one hundred and twenty 6-week-old carrier pigeons were obtained from two private breeding facilities. In the first breeding facility (A), there has been no YPDS history for the last 5 years, whereas in the second one (B) problems with YPDS have been noted each year. Before the experiment, cloacal swabs and blood samples were collected from all birds originating from each flock to rule out or confirm PiCV infection with the qPCR method described previously [20] and to determine the presence of antibodies against PiCV with the in-house ELISA method developed in the previous study [19]. The pigeons were also screened for pigeon herpesvirus, pigeon adenovirus, *Chlamydia psittaci*, *Salmonella* spp., and endoparasites, and they were negative. The birds were housed in isolated units in a PCL3 biosafety facility of the Department of Poultry Diseases, Faculty of Veterinary Medicine of the University of Warmia and Mazury in Olsztyn. The biosafety facility is equipped with an HEPA filtering system and an automated system for pressure control in corridors, bird units, and hygiene stations to prevent contamination of experimental premises. Every group of pigeons was housed in a separate unit. The birds were administered seed mixtures and water ad libitum throughout the experiment.

### Experimental design

Pigeons were divided into 4 groups, 30 birds each. Groups V and C consisted of healthy pigeons, negative for PiCV genetic material and for antibodies against this virus, originating from the breeding facility A, whereas groups V1 and C1 were composed of clinically healthy birds, negative for antibodies against PiCV but positive for this virus in qPCR screening (naturally infected pigeons), originating from the breeding facility B. After two weeks of the adaptation period, the pigeons from groups V and V1 were vaccinated with PiCV rCP mixed with an adjuvant, whereas the birds from groups C and C1 were controls (immunized with an adjuvant only). The birds were vaccinated again 21 days after the first immunization (35^th^ day of the experiment). On the day of the first vaccination and 2, 23, 39 and 46 days post first vaccination (dpv), blood samples were collected from six birds of each group. Next, those pigeons were euthanized and their spleen samples were collected during an anatomopathological examination. Blood samples were used to obtain sera for determination of anti-PiCV rCP antibodies with in-house ELISA and for total DNA extraction to evaluate PiCV viral loads with droplet digital PCR (ddPCR). From the spleen samples, mononuclear cells were isolated and divided into two parts: one part was used for determination of anti-PiCV rCP IgY-secreting B cells (SBC) with ELISPOT (2–46 dpv) and the second one was used for RNA extraction to evaluate the expression of genes encoding CD4 and CD8 T cells surface receptors and IFN-γ (0–46 dpv).

### Isolation of mononuclear cells

Mononuclear cells from the whole spleens collected from each bird were isolated with the method described previously [5]. Cell concentrations and the percentage of viable cells were determined in the Vi-cell XR analyzer (Beckman Coulter, USA).

### RNA isolation and qPCR for CD4, CD8, and IFN-γ gene expression

The number of mononuclear cells isolated from spleen samples was standardized to 5 x 10^6^ and used for RNA isolation with the use of the RNeasy Mini Kit (Qiagen, Germany) according to the manufacturer’s protocol. Genomic DNA remaining in the samples after RNA isolation was digested with deoxyribonuclease I (Sigma Aldrich, USA). The concentration of eluted RNA was measured with a NanoDrop 2000 spectrophotometer (Thermo Fisher Scientific, USA), and the samples were stored at -80°C until further analyzed.

Reverse transcription was carried out with the High-Capacity cDNA Reverse Transcription Kit (Life Technologies, USA) according to the manufacturer’s recommendations. The concentration of RNA was standardized to 0.5 μg per sample. The relative expression of the genes encoding CD4 and CD8 T cells receptors and IFN-γ was determined by qPCR method described previously [21] with the use of Power SYBR Green PCR Master Mix kit (Life Technologies, USA) and LightCycler 96 (Roche, Switzerland).

### ELISPOT for determination of anti-PiCV rCP Ig Y-SBC

The MultiScreen plates (Merck Millipore, USA) were coated with 100μL/ well of a PBS solution of PiCV rCP (conc. 20μg/ 1 mL). The samples were standardized to 1.5 x 10^5^ mononuclear cells and seeded in triplicate directly to the wells of previously coated with PiCV rCP plates. After incubation with Iscove’s modified Dulbecos medium (IMDM; Sigma Aldrich, Germany), the plates were rinsed and incubated overnight with biotinylated rabbit anti-pigeon IgY (Antibodies-online, USA). After rinsing, 100 μL of streptavidin, alkaline phosphatase (S-AP) (Vector Laboratories, USA) dissolved 1:500 in PBS (Sigma Aldrich, Germany) were added to each well. Enzymatic reaction was performed with an alkaline phosphatase substrate (BCIP/NBT; Vector Laboratories, USA) and stopped with distilled water. All details concerning rinsing and incubation steps were described in our previous study [5]. Counting of spot forming units (SFU) corresponding to anti-PiCV rCP IgY-SBC was performed with an Eli.Scan plate scanner and Eli.Analyse software (A-EL-VIS, Germany). Data were expressed as the mean absolute number of SFU +/- standard deviation per 1 x 10^6^ of mononuclear cells in each group in each day of sampling.

### In-house ELISA for determination of anti-PiCV rCP IgY

The assay was performed according to the protocol described in the previous study [19]. The wells of Nunc-Immuno Module plates (Thermo Scientific, USA) were coated with 100 μL of PiCV rCP (concentration 20 μg/ mL). Pigeon sera were diluted at 1:400. The rabbit anti-pigeon IgG (dilution 1:30 000; Antibodies-online, USA) were used as primary antibodies. The goat anti-rabbit IgG with horseradish peroxidase (HRP) (dilution 1:1 000; BD biosciences, USA), were used as secondary antibodies. Each rinsing step was performed with an ELx 405 automatic washer (Biotek, USA). Optical density was measured with an ELx 800 spectrophotometer (Biotek, USA) at the wavelength of 450 nm. Data were expressed as mean OD_450_ +/- standard deviation in each group in each sampling.

### Detection of PiCV viral loads in serum samples with ddPCR

The droplet digital PCR (ddPCR) was performed to detect PiCV viral loads in serum samples at all sampling dates. The genomic DNA was extracted from 200 μL of the serum using a DNeasy Blood & Tissue Kit (Qiagen, Germany) in accordance with the manufacturer’s instructions. The ddPCR reaction mixture contained 10 μL of QX200 ddPCR EvaGreen Supermix (Bio-Rad, USA), 0.4 μL of the forward primer (10 μM), 0.2 μL of the reverse primer (10 μL), and 4.54 μL of template DNA, and was topped up to the volume of 20 μL with nuclease-free water. The primer sequences were designed previously [20]. The reaction mixture was transferred into individual wells of a disposable eight-channel DG8 cartridge (Bio-Rad, USA) that had been already preloaded in a DG8 cartridge holder (Bio-Rad, USA) and the bottom wells were filled with 70 μL of droplet generation oil. The prepared cartridge was then placed into the QX 200 droplet generator (Bio-Rad, USA). The prepared droplet emulsions were further loaded into a semi-skirted, PCR-clean 96-well plate (Bio-Rad, USA) using a multichannel pipette, by aspirating 40 μL from the DG8 cartridge. The plate was then heat-sealed with pierceable foil using a PX1 PCR plate sealer (Bio-Rad, USA), and PCR amplification was carried out in a C1000 Touch thermal cycler (Bio-Rad, USA). The thermal cycling consisted of initial denaturation at 95°C for 5 min followed by 40 cycles of 95°C for 30 s, and 64°C for 1 min with a ramp rate of 2°C/s; the next cycle was performed at 4°C for 5 min followed by 90°C for 5 min. After thermal cycling, the plate containing the droplets was placed in a QX 200 droplet reader (Bio-Rad, USA) for analysis. Each sample was analyzed in duplicate, and results were calculated with the following formula: mean number of positive droplets in the sample minus mean number of the positive droplets in the negative control. The results was expressed as mean PiCV genome copy number +/- standard deviation per 20 μL of sample in each group in each day of sampling.

### Statistical analysis

The significance of differences in values of the measured parameters between the investigated groups was analyzed with the Kruskal-Wallis non-parametric test for independent samples using Statistica.pl v.10.0 software. Differences were considered significant at confidence levels of 95 and 99% (p< 0.05 and p<0.01, respectively).

## Results

### CD4, CD8, and IFN-γ gene expression

**Fig 1:**
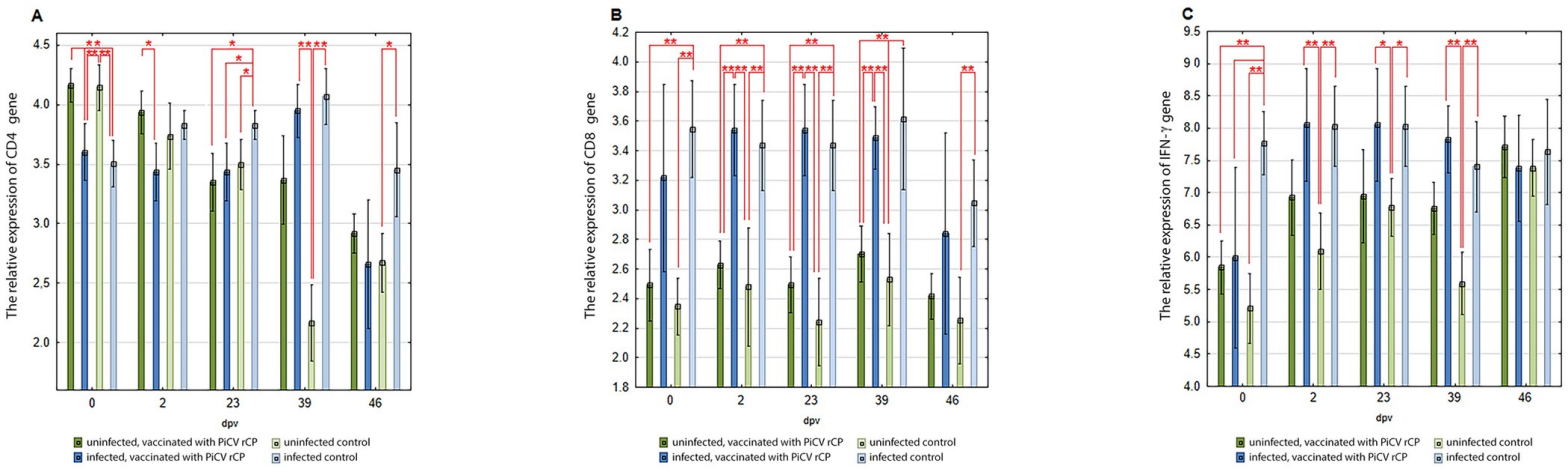
Mean relative expression of the genes encoding CD4 receptor (A), CD8 receptor (B), and IFN-γ (C) in splenic mononuclear cells of investigated pigeons. The pigeons were vaccinated with PiCV rCP mixed with adjuvant (groups V and V1) or adjuvant only (groups C and C1) on day 0 and boosted on day 21. Groups V1 and C1 consisted of birds asymptomatically infected with PiCV, whereas groups V and C consisted of uninfected pigeons. Samples were collected from pigeons of all groups on days 0, 2, 23, 39, and 46 after first vaccination (dpv). The asterisks indicate a statistically significant difference between investigated groups, where * p < 0.05, ** p < 0.01 in Kruskal-Wallis non-parametric test for independent samples. Error bars represent the standard error of the mean.

The expression of CD4 gene in uninfected pigeons was not significantly higher in the PiCV rCP immunized birds than in the control group almost through the entire post-vaccination period. An opposite trend was observed in groups of naturally infected pigeons. A comparison of infected and uninfected groups of birds showed that the CD4 gene expression was significantly higher in birds from V and C groups than in V1 and C1 pigeons before the first immunization (p = 0.00). On 2dpv, the expression of this gene was higher in group V than in group V1 (p = 0.01). The expression of CD4 gene was higher in group C1 than in all other groups on 23 dpv (p = 0.01 to 0.03) and was higher than in C group pigeons on 39 and 46 dpv (p = 0.00 and 0.01, respectively). On 39 dpv, the expression of this gene in group C was significantly lower than in group V1 (p = 0.00) (Fig 1A).

The expression of CD8 gene was higher in the uninfected and immunized with PiCV rCP pigeons than in the control birds in all sampling dates, but the difference was not statistically significant. A similar tendency was not observed in both groups of naturally infected pigeons. A comparison of both uninfected and infected with PiCV groups of pigeons showed that the expression of CD8 gene was higher in V1 and C1 group pigeons than in V and C group birds throughout the experiment, but differences were significant only on 2–23 dpv (p = 0.00). The significant differences (p = 0.00) were also noted between group C1 and groups C and V on the day of immunization and on 46 dpv (Fig 1B).

The expression of IFN-γ gene was not significantly higher in the uninfected and immunized with PiCV rCP pigeons than in the control group C birds almost through the entire experiment. No similar trend was reported in the pigeons naturally infected with PiCV. A comparison of both uninfected and infected with PiCV groups of birds showed that the expression of IFN-γ gene was higher in C1 group pigeons than in all other groups of birds on the day of the first immunization (p = 0.00 to 0.04). The expression of this gene was also higher in pigeons from groups V1 and C1 than in C group birds on 2–39 dpv (p = 0.00 to 0.04) (Fig 1C).

### anti-PiCV rCP IgY-SBC

**Fig 2:**
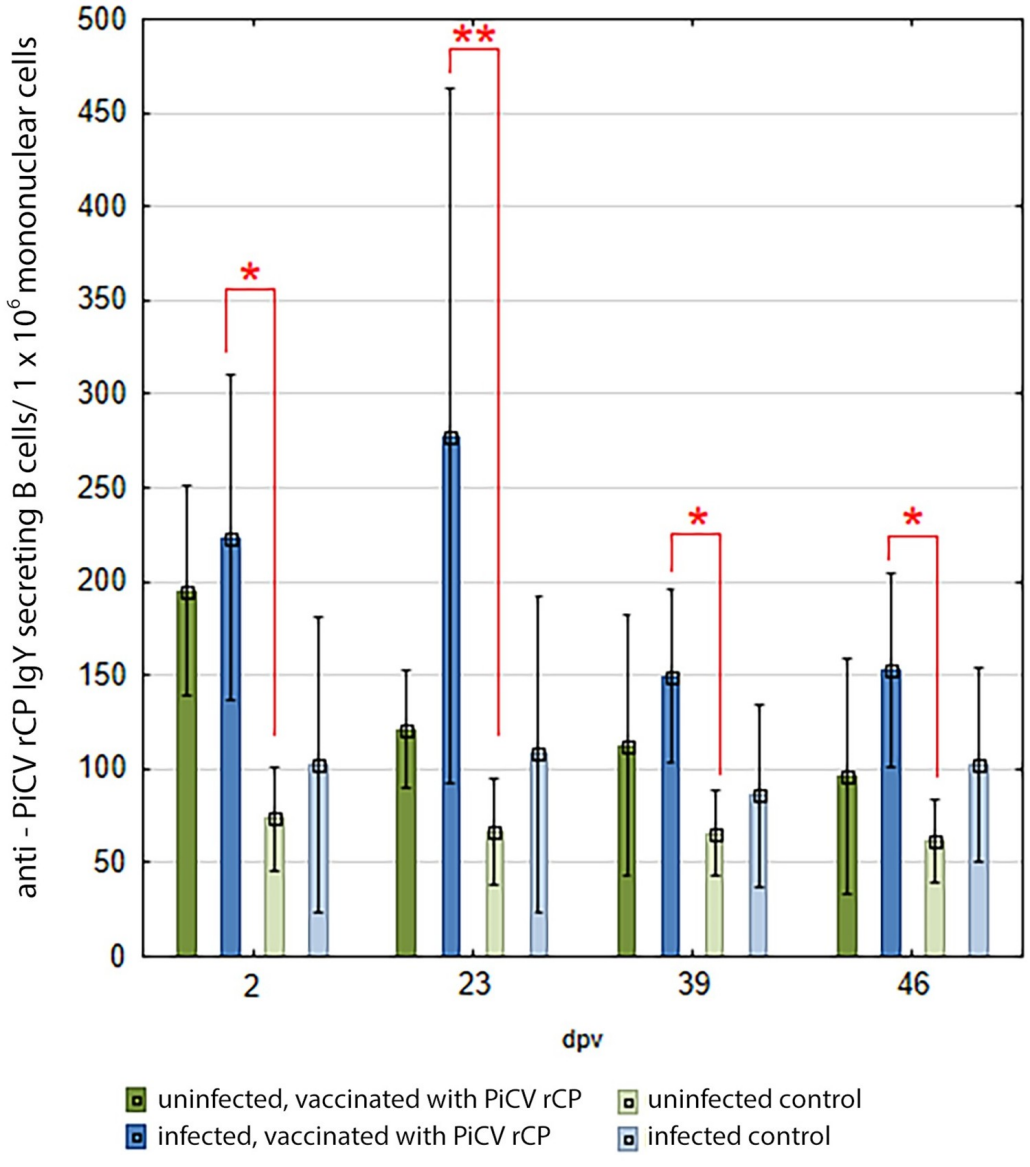
The results of ELISPOT for anti-PiCV rCP IgY–SBC in the spleens of the investigated pigeons. The pigeons were vaccinated with PiCV rCP mixed with adjuvant (groups V and V1) or adjuvant only (groups C and C1) on day 0 and boosted on day 21. Groups V1 and C1 consisted of birds asymptomatically infected with PiCV, whereas groups V and C consisted of uninfected pigeons. Samples were collected from pigeons of all groups on days 2, 23, 39, and 46 after first vaccination (dpv). The asterisks indicate a statistically significant difference between investigated groups, where * p < 0.05, ** p < 0.01 in Kruskal-Wallis non-parametric test for independent samples. Error bars represent the standard error of the mean.

The number of anti-PiCV IgY secreting B cells was higher in the pigeons immunized with PiCV rCP than in the control groups in both infected and uninfected pigeons on all dpv, but the differences were significant only between groups V1 and C (p = 0.00 to 0.03). The SFU number in group V was the highest on 2 dpv (approx. 200.0 +/-53.0 per 1 x 10^6^ cells) and gradually decreased to approx. 100.0 +/- 60.0 per 1 x 10^6^ cells on 46 dpv. Whereas in group V1, the anti-PiCV rCP IgY-SBC number was the highest on 23 dpv (approx. 280 +/- 176.56 per 1 x 10^6^) and decreased to approximately 150.0 +/- 45.0 per 1 x 10^6^ cells on 39 and 46 dpv (Fig 2). In control groups, the anti-PiCV rCP IgY-SBC number was constant throughout the experiment and reached 67.0 +/- 24.0 and 99.0 +/- 63.0 per 1 x 10^6^ cells on average in groups C and C1, respectively.

### anti-PiCV rCP IgY

**Fig 3:**
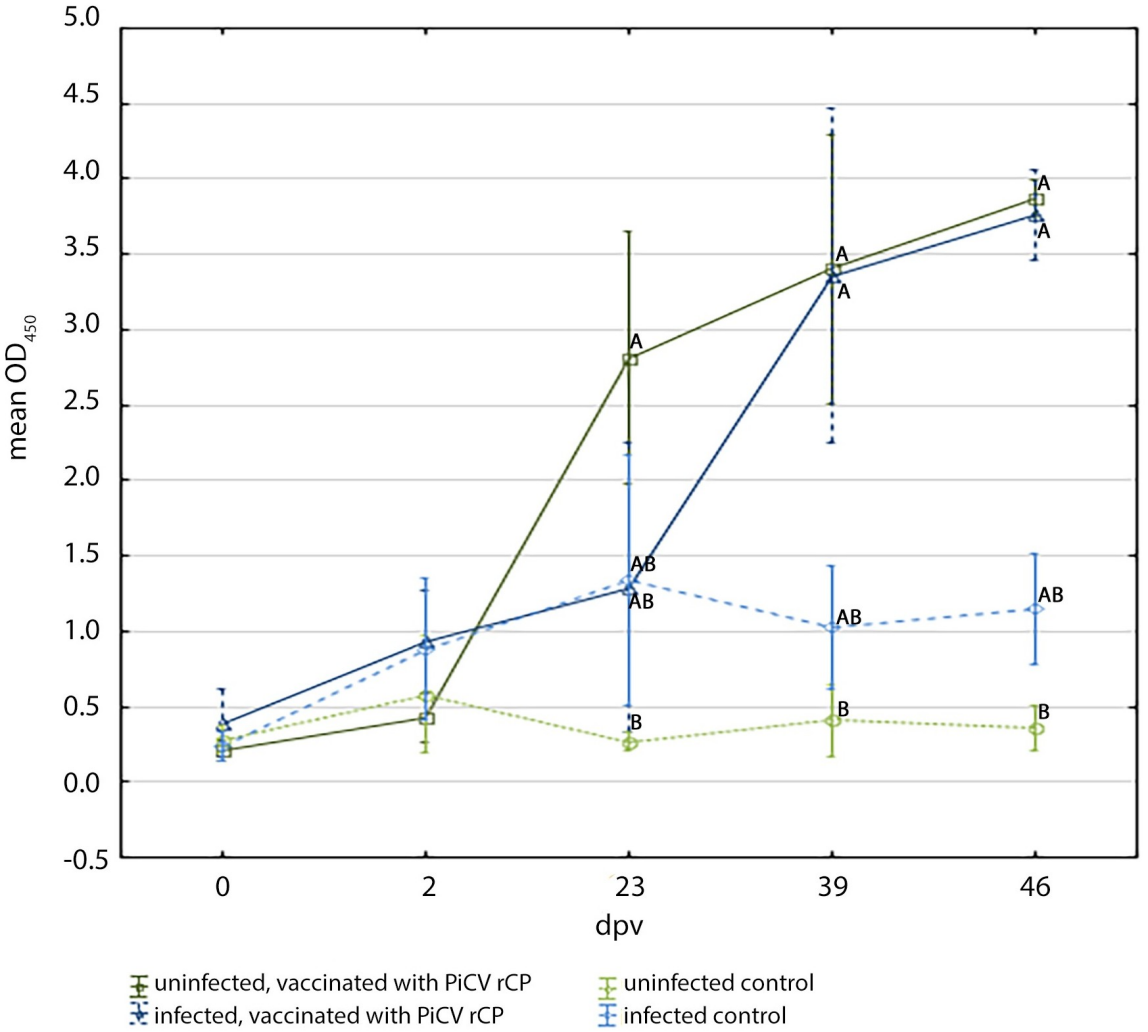
Detection of PiCV rCP–specific IgY in sera of the examined pigeons using in-house ELISA. The pigeons were vaccinated with PiCV rCP mixed with adjuvant (groups V and V1) or adjuvant only (groups C and C1) on day 0 and boosted on day 21. Groups V1 and C1 consisted of birds asymptomatically infected with PiCV, whereas groups V and C consisted of uninfected pigeons. Samples were collected from pigeons of all groups on days 2, 23, 39, and 46 after first vaccination (dpv). The different letters (A,B) between the group symbols indicate a statistically significant difference between investigated groups (p < 0.01) in certain sampling date in Kruskal-Wallis non-parametric test for independent samples. Error bars represent the standard error of the mean.

The pigeons from all investigated groups had low OD_450_ values before the immunization (0.27 +/- 0.15 on average). A significant increase of anti-PiCV rCP IgY was noted on 23 dpv in group V pigeons (OD_450_ = 2.8 +/- 0.8) and was significantly higher than in group C birds (p = 0.00). Whereas the OD_450_ value in group V1 pigeons on 23 dpv reached 1.28+/- 0.91 and was similar to that determined for C1 birds (OD_450_ = 1.33 +/- 0.78). On 39 and 46 dpv, the OD_450_ value achieved similar levels (approx. 3.5 +/- 0.8 to 4.0 +/- 0.2) in groups V and V1 and was significantly higher (p = 0.00) than in the control group C (Fig 3).

### PiCV viral loads in serum samples

**Fig 4:**
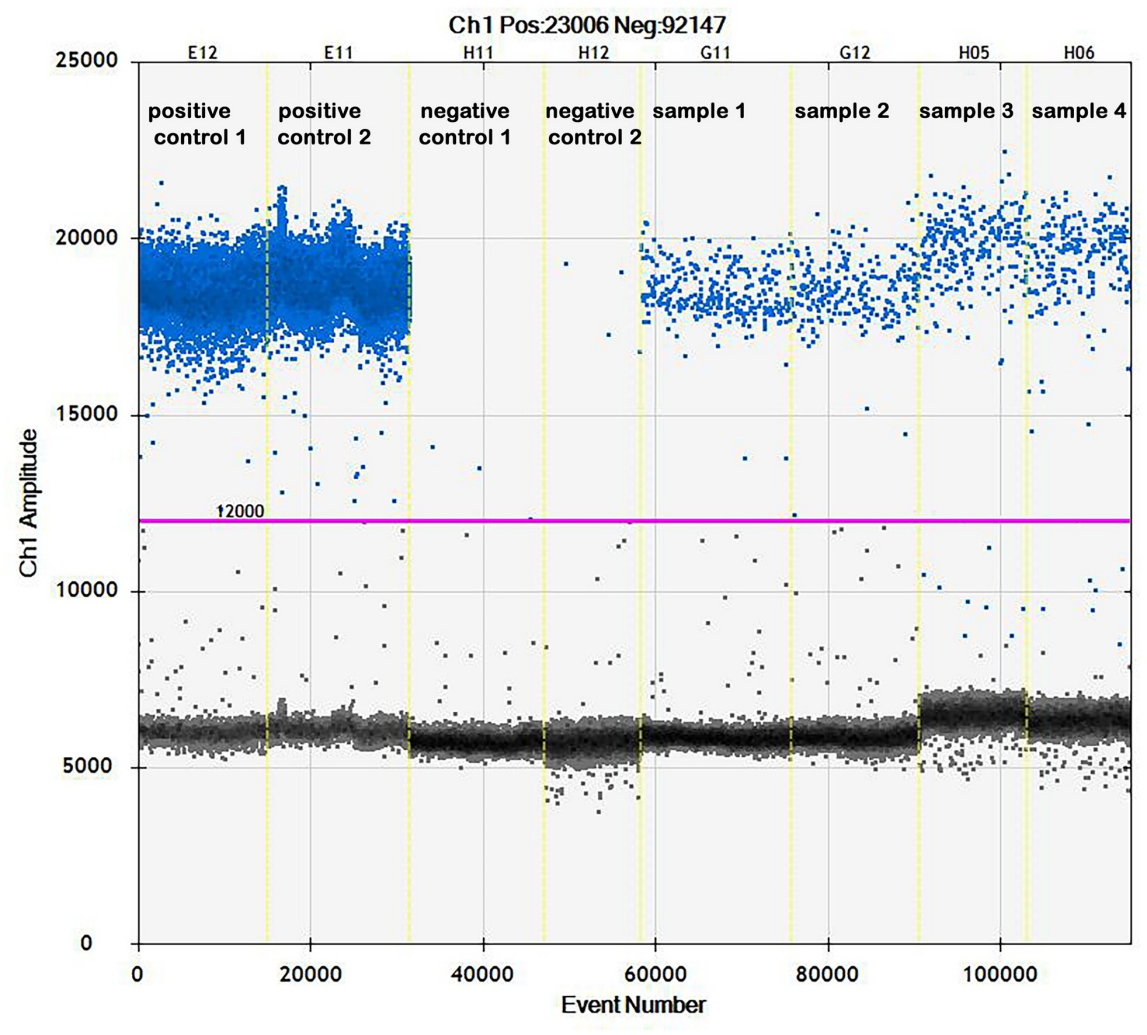
One dimensional plot of ddPCR assay showing PiCV viral loads in selected samples. The samples are divided by vertical dotted yellow lines. The unbroken pink line is the threshold, above which there are positive droplets (blue) with PCR amplification and underneath which there are negative droplets (gray) without any amplification.

Exemplary results of ddPCR are presented in Fig 4. All pigeons from groups V1 and C1 were positive for PiCV genetic material throughout the experiment (except V1 group birds on 39 dpv where 2 samples were detected as negative). The PiCV viral loads were the highest on the day of the first vaccination in the sera of pigeons from both naturally infected groups (203851.2 +/-53332.3 and 216327.1 +/- 17645.77 for V1 and C1 groups, respectively) and decreased gradually to 1.62 +/- 2.13 (V1 group) and 9.82 +/- 9.88 (C1 group) on 39 dpv. On 46 dpv, the PiCV viral loads increased slightly in the sera of pigeons from both naturally infected with PiCV groups. The PiCV viral loads were higher in C1 than in V1 group pigeons throughout the experiment, but the biggest difference was noted on 23 dpv. There was no presence of PiCV genetic material in the sera of pigeons from both uninfected groups (Table 1).

**Table 1:** The PiCV viral loads in the sera of investigated pigeons.

Group		The PiCV viral loads					The number of positive samples				
		days post first vaccination					days post first vaccination				
		0	2	23	39	46	0	2	23	39	46
Uninfected vaccinated with PiCV rCP	x¯ SD	00.00 00.00	00.00 00.00	00.00 00.00	00.00 00.00	00.00 00.00	0/6	0/6	0/6	0/6	0/6
Infected vaccinated with PiCV rCP	x¯ SD	203851.2 53332.3	351.96 164.23	30.8 26.11	1.62 2.13	6.62 2.13	6/6	6/6	6/6	4/6	6/6
Uninfected control	x¯ SD	00.00 00.00	00.00 00.00	00.00 00.00	00.00 00.00	00.00 00.00	0/6	0/6	0/6	0/6	0/6
Infected control	x¯ SD	216327.1 17645.77	368.89 25.85	336.93 320.41	9.82 9.88	16.64 13.50	6/6	6/6	6/6	6/6	6/6

## Discussion

Because PiCV is thought to be one of the factors involved in YPDS and is very prevalent in pigeons, the development of effective vaccine against this virus could be a strategy to control the YPDS. It was demonstrated that both humoral and cellular compartments of the immunity are apparently involved in the immune response developed upon PiCV rCP immunization [5], but nothing is known about the immune response to this antigen in pigeons with different PiCV infectious status.

The interesting data about cell-mediated immune response to various factors could be provided by the evaluation of the expression of genes encoding T lymphocyte receptors or IFN-γ. In the previous basic research, no significant differences were observed in the percentage of T CD4 and CD8 lymphocytes in the blood and spleens between immunized with PiCV rCP and unimmunized pigeons [5]. However, in the immunized pigeons, the percentage of T CD4^+^ lymphocytes was not significantly higher than in the unimmunized control birds, whereas the percentage of T CD8^+^ lymphocytes was usually similar to that found in the control group. In the current investigation, we employed a molecular method to determine the expression of genes encoding CD4 and CD8 surface receptors of T lymphocytes. One of the previous studies revealed that this method could provide reliable results and can be used as an alternative to flow cytometry [21]. In the present research, the CD4 and CD8 gene expression was higher in mononuclear cells isolated from the spleen of uninfected with PiCV and immunized with PiCV rCP pigeons than in the control group C birds during almost the whole post-vaccination period, but the differences were not statistically significant. These results partially correspond with findings from our previous basic investigation performed on uninfected with PiCV pigeons [5]. Interestingly, this tendency was not observed in the case of pigeons naturally infected with PiCV. In the case of these birds, the CD4 gene expression was usually lower in naturally infected and immunized with PiCV pigeons than in the control group C1 birds, whereas no tendency was observed in the case of CD8 gene expression. The differences between both groups of naturally infected with PiCV pigeons were not statistically significant. However, numerous significant differences were noted between both naturally infected with PiCV pigeons and both uninfected groups of birds, which was clearly visible in the case of CD8 gene expression. In the case of CD4 gene expression, this trend was noted only on 39 and 46 dpv. Partially similar results were achieved by Ferrari et al. [22] who noted higher levels of CD4^+^ and CD8^+^ lymphocytes in piglets vaccinated with PCV2 rCP and experimentally challenged with this virus. The significantly higher CD8 gene expression in pigeons naturally infected with PiCV than in the uninfected birds, with no differences between vaccinated and unimmunized birds, suggests that it resulted from the response to natural infection with the PiCV, and not from the vaccination. In the light of the above, the immunization with PiCV rCP has no influence on the expression of this gene despite PiCV infectious status.

The higher production of IFN-γ as a response to immunization with the use of circovirus recombinant capsid protein was broadly described in pigs [22] and recently in pigeons [5]. The results of our investigation showed only a trend for higher expression of this gene in mononuclear cells isolated from the spleen of uninfected and immunized with PiCV rCP pigeons. Interestingly, the pattern of IFN-γ gene expression was similar to the CD8 gene expression pattern, especially in the case of naturally infected pigeons. The ddPCR used in this study for the detection of PiCV viral loads in serum samples allowed detecting even samples with very small amounts of PiCV genetic material, which increased the reliability of the results obtained. The presence of PiCV genetic material in the sera of both naturally infected groups of pigeons confirms the viremia. It also confirms that the increased expression of CD8 and IFN -γ genes was due to the viral infection, and not to the vaccination. The results of ddPCR showed that the PiCV viral loads decreased gradually till 39 dpv and increased slightly on 46 dpv, which was convergent with the suppressed expression of CD8 and IFN-γ genes in the last sampling date in both groups of naturally infected pigeons. This data is also in accordance with the results obtained by Ferrari et al. [22], who noted an increase in IFN-γ productivity in some pigs vaccinated with PCV2 rCP after challenge with PCV2.

The measurement of the specific humoral immune response is always a useful tool allowing the evaluation of the immunogenicity of antigens. In the current study, we detected differences in the time of appearance of anti-PiCV rCP IgY-SBC between pigeons naturally infected and uninfected with PiCV. In the uninfected pigeons, the highest number of secreting specific antibody B cells was noted on 2dpv, which is consistent with results of our previous study [5], whereas in the naturally infected birds the number of anti-PiCV rCP IgY–SBC was high on 2 dpv, but peaked on 23 dpv. The reason for the later peak of secreting specific antibody B cells number in the infected with PiCV than in the uninfected pigeons, could result from the suppression of humoral immunity caused by the circovirus. These results correspond to the IgY specific immune response measured by in-house ELISA. The results of in-house ELISA showed that the level of anti- PiCV rCP IgY increased successively in both groups of vaccinated pigeons, but seroconversion was detected earlier in the uninfected birds (23 dpv). Whereas in both groups of naturally infected pigeons, the OD_450_ value on 23 dpv was similar and not significantly higher than before the experiment. The slight increase of antibodies in both groups of birds naturally infected with PiCV may be related to the subclinical infection with this virus. However, no further increase of OD_450_ value was observed in C1 group pigeons. Whereas in the case of the birds naturally infected with pigeon circovirus and immunized with PiCV rCP, a significant increase was observed in the OD_450_ value since 39 dpv. At the end of the experiment, antibody levels were very similar in both vaccinated groups of birds independently from their PiCV infectious status, which suggests that the immunization of infected pigeons could be effective. In this study, a reverse dependency was noticed between levels of anti-PiCV rCP IgY in pigeons sera and anti-PiCV rCP IgY-SBC number in the spleen. The anti-PiCV rCP IgY levels increased successively during the experiment to reach the highest value on 46 dpv, whereas the number of anti-PiCV rCP IgY- SBC decreased successively in each sampling in V group pigeons and decreased since 23 dpv in V1 group birds. This tendency is typical of immunization and viral infections, as described earlier [5, 23]. The kinetics of anti-PiCV rCP IgY-SBC and antibody response to PiCV rCP indicates that the specific humoral immune response in asymptomatically infected with PiCV pigeons appears later than in the uninfected birds. This suggests the immunosuppressive effect of PiCV on humoral component of the immunity. Previous research showed that PiCV infection led to apoptosis induction in lymphocytes present in the bursa of Fabricius [7]. The degradation of bursal lymphocytes leads to the suppression of humoral immunity, which explains the phenomenon noticed in our study. The depletion of B lymphocytes was also described in the case of PCV2 infection [24].

Pigeon circoviruses are characterized by a high genetic diversity reaching even up to 20% [12–15, 17, 18, 25, 26], which today allows separating as many as 6 different clades of this virus denoted with letters from A to F [25, 26]. They key fragment of the PiCV genome is the capside protein encoding gene, and its variability is reflected in the affiliation of viruses to specified clades, even in analyzing the full genomes of this virus [13]. This genetic diversity may also affect antigene diversity and thereby also the specificity of the immune response to vaccination with PiCV rCP against viruses representing various genotypes. It is extremely difficult to predict which virus genotype would cause infections of pigeons in the field conditions, because international trade of these birds and competition flights facilitate the long-distance spread of infections with different strains. Of all known PiCV genotypes, the genotype A is the most widespread in Europe and Asia. The other genotypes are detected significantly less often [15, 18, 25, 26]. For this reason, we have chosen a representative of genotype A for vaccine development. The homology of the CP amino acid sequence of most isolates belonging to this genotype is 99% on average. Because we had no access to pigeons naturally infected with viruses representing other genotypes, our experiment was conducted with birds infected with PiCV belonging to genotype A, but differing significantly in the *cap* sequence from the virus used to develop a subunit vaccine (88% and 92% nucleotide and amino acid homology, respectively). Our experiment demonstrated that the pigeons infected with the PiCV isolate representing a genotype homologous with the vaccine responded correctly to vaccination.

Scientific literature lacks data on the immune response of pigeons to infections with various PiCV strains. Due to multiple similarities in the immunopathogenesis between PiCV infections and porcine circovirus type 2 (PCV2), comparative analysis can be undertaken, as it had been done before [5]. The PCV2 occurring in pigs is also characterized by a high variability of the sequence of the capsid protein encoding gene, and, like in the case of PiCV, this variability allowed distinguishing a few genotypes denoted with letters a-d. Initially, the PCV2a was the prevailing genotype, but introduction of vaccinations against this genotype reduced its prevalence in favor of genotype PCV2b which has been successively replacing it [27]. A similar course of events may be expected during pigeons immunization with the vaccine based on one PiCV genotype. Investigations conducted across the globe have demonstrated, however, that immunization of pigs with vaccines based on PCV2a genotype tuned out protective against PCV2b [28, 29]. Nevertheless, the emergence of another genotypes of this virus (like PCV2d) contributed to the reduced efficacy of vaccinations. The amino acid homology of capsid protein of the pigeon circovirus belonging to genotype A with other genotypes of this virus reaches 85% for genotypes B and C, 77% for genotype D, 74% for genotype E, and barely 67% for genotype F [13].

Summarizing the results of this investigation, it could be stated that the immune response of pigeons to the PiCV rCP varies depending on PiCV infectious status. The cell mediated immune response to infection appears faster, but it is visible only as a trend for higher expression of genes encoding IFN-γ and T-lymphocyte receptors. The lack of statistical differences between both infected groups shows that PiCV infection masks the potential cellular immune response to the vaccination with PiCV rCP. The specific humoral immune response was developed in both uninfected and infected pigeons, but appeared later in the subclinically infected individuals, probably due to the immunosuppressive effect of the PiCV. The introduction of a vaccine based on one prevailing PiCV genotype only may lead to partial inefficacy of vaccinations in the case of the other genotypes, which will enable their greater expansion.

## Conclusions

In the light of the obtained results, it could be concluded that pigeon circovirus recombinant capsid protein elicits the immune response in both naturally infected and uninfected pigeons, but the rate of immune response varies depending on PiCV infectious status. Further investigation involving experimental infection with PiCV in the PiCV rCP-vaccinated pigeons should be conducted to evaluate if the stimulatory effect of PiCV rCP would be efficient in reducing virus replication, virus shedding, and development of YPDS. Considering the high variability of PiCV, noteworthy is the implementation of multi-genotype vaccines.

## Supporting information

S1 Fig Genetic affiliation and homology of the amino acid and nucleotide sequence of the PiCV isolated from experimental birds and used for vaccine development. A. A neighbor-joining phylogenetic tree depicting the possible evolutionary relationships between PiCV sequences from GenBank and PiCV isolates occurring in experimental birds and the isolate used for vaccine preparation. The clades of PiCVs are marked with the following colours: A—pink, B—dark green, C—navy blue, D—brown, E—grey, and F—black. The isolate used as a vaccine is marked with red, the strains isolated from birds considered for the experiment are marked with purple (not used for the reason of high homology) and orange (used for the experiment). B. Pairwise identity matrices calculated with the Clustal W method comparing sequence of vaccine isolate and isolates occurring in birds considered for the experiment. Based on this analysis, the birds infected with isolate PiCV_2T were selected for the experiment. The percentage of nucleotide identity of *cap* is presented in bottom left and the percentage of amino acids identity in protein encoded by this gene is presented in top right.
(TIF)
