# Supplementary figures and images for: Comparison of the immune response to vaccination with pigeon circovirus recombinant capsid protein (PiCV rCP) in pigeons uninfected and subclinically infected with PiCV
Source: PLoS One. 2019 Jun 28;14(6):e0219175. doi: 10.1371/journal.pone.0219175 (PMC6599111; doi:10.1371/journal.pone.0219175)

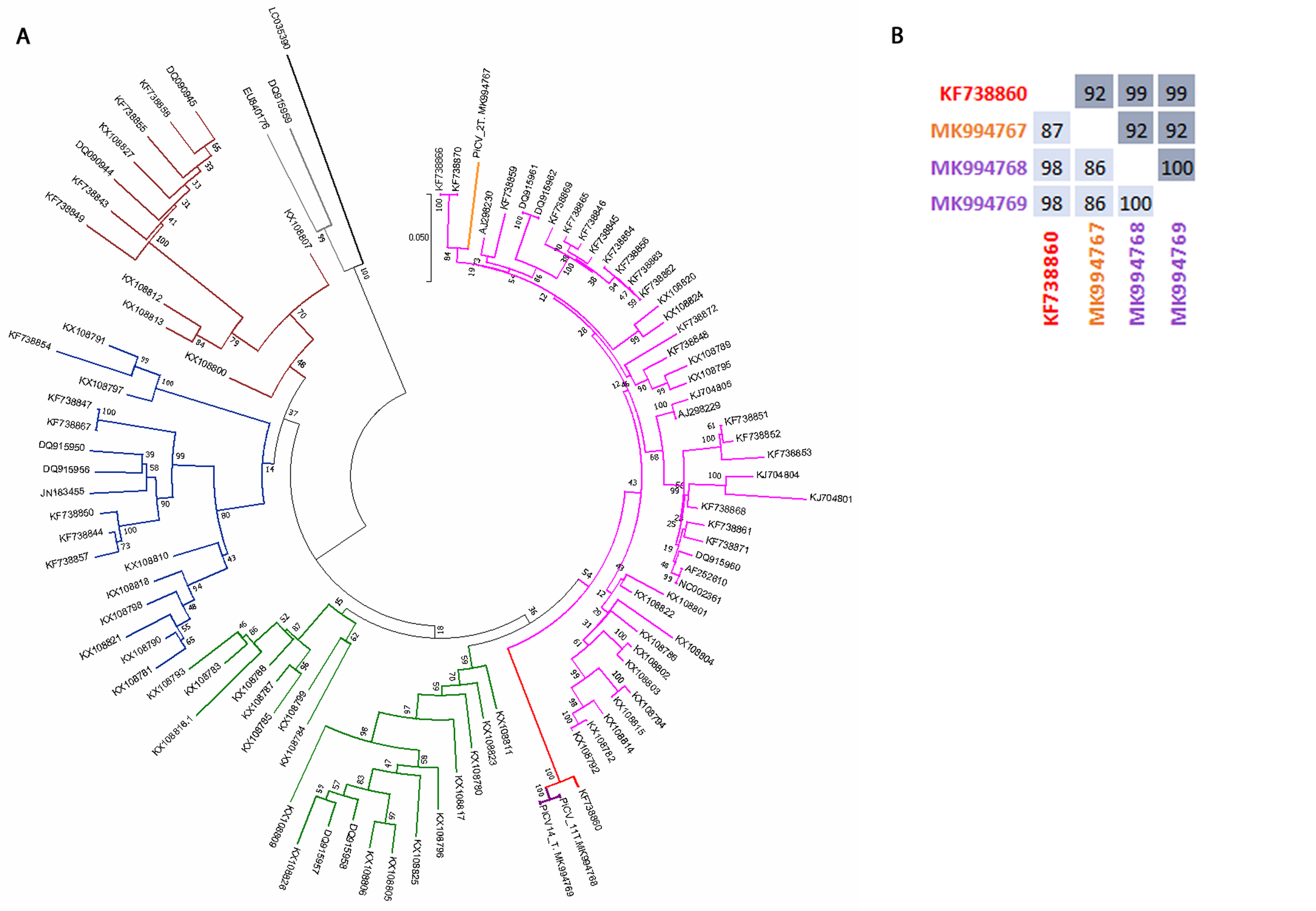

Supplement: S1 Fig — A. A neighbor-joining phylogenetic tree depicting the possible evolutionary relationships between PiCV sequences from GenBank and PiCV isolates occurring in experimental birds and the isolate used for vaccine preparation. The clades of PiCVs are marked with the following colours: A—pink, B—dark green, C—navy blue, D—brown, E—grey, and F—black. The isolate used as a vaccine is marked with red, the strains isolated from birds considered for the experiment are marked with purple (not used for the reason of high homology) and orange (used for the experiment). B. Pairwise identity matrices calculated with the Clustal W method comparing sequence of vaccine isolate and isolates occurring in birds considered for the experiment. Based on this analysis, the birds infected with isolate PiCV_2T were selected for the experiment. The percentage of nucleotide identity of cap is presented in bottom left and the percentage of amino acids identity in protein encoded by this gene is presented in top right. (TIF) [file pone.0219175.s001.tif]
